# Differentiation of autism spectrum disorder and mood or anxiety disorder

**DOI:** 10.1177/13623613211039673

**Published:** 2021-08-18

**Authors:** Sarah Wittkopf, Sanna Stroth, Anika Langmann, Nicole Wolff, Veit Roessner, Stefan Roepke, Luise Poustka, Inge Kamp-Becker

**Affiliations:** 1 Philipps-University Marburg, Germany; 2 TU Dresden, Germany; 3 Charité – Universitätsmedizin Berlin, Germany; 4 University Medical Center Göttingen, Germany

**Keywords:** anxiety disorders, Autism Diagnostic Interview–Revised, Autism Diagnostic Observation Schedule, autism spectrum disorder, mood disorder

## Abstract

Autism spectrum disorder shares many symptoms with other mental health disorders, and comorbid disorders such as mood and anxiety disorders are common, making the diagnostic process challenging. We aimed to explore the diagnostic accuracy of two standard autism spectrum disorder diagnostic instruments and to identify those behavioral items that best differentiate between autism spectrum disorder and mood and anxiety disorder in a naturalistic sample of patients utilizing autism spectrum disorder specialist services. The study included data of 847 participants (5–65 years of age, *n* = 586 with autism spectrum disorder, *n* = 261 with mood and anxiety disorder) all evaluated with the Autism Diagnostic Observation Schedule in the context of the diagnostic process. Data of the Autism Diagnostic Interview–Revised were available for 428 participants (5–51 years of age, *n* = 367 with autism spectrum disorder, *n* = 61 with mood and anxiety disorder). By means of binominal logistic regressions and an ensemble feature selection, we identified a subset of items that best differentiated between autism spectrum disorder and mood and anxiety disorder. Overall, the results indicate that a combination of communicational deficits and unusual and/or inappropriate social overtures differentiates autism spectrum disorder and mood and anxiety disorder. Aspects of social cognition are also relevant. Limitations of the current study and implications for research and practice are discussed.

**Lay abstract:**

Symptoms of mood and anxiety disorders overlap with symptoms of autism spectrum disorder, making the diagnostic process challenging. This study found that a combination of communicational deficits and unusual and/or inappropriate social overtures facilitates differentiation between autism spectrum disorder and mood and anxiety disorders. Furthermore, the results confirm the essential need of a behavioral observation with the Autism Diagnostic Observation Schedule in combination with a full Autism Diagnostic Interview–Revised to support diagnostic decisions.

## Introduction

Autism spectrum disorder (ASD) is a neurodevelopmental disorder with a worldwide prevalence of just less than 1% (Lord et al., 2020). Comorbid disorders, including affective, anxiety, and emotional disorders as well as attention deficit hyperactivity disorder (ADHD) and personality disorders, are common in individuals with ASD (Gjevik et al., 2011; Joshi et al., 2013; Simonoff et al., 2008). Prevalence rates of anxiety disorders in young individuals with ASD are nearly 40% (van Steensel et al., 2011) and higher compared with those found in typically developing children. In adults, prevalence rates are even higher—up to 84% (Postorino et al., 2017). Furthermore, depressive disorders are highly prevalent in individuals with ASD. According to a recent meta-analysis, there is a lifetime prevalence of 14.4% and a current prevalence of 12.3% for depressive disorders in ASD patients (Hudson et al., 2019).

Besides high rates of comorbidities, the differentiation of ASD versus mood or anxiety disorder (MAD) can be difﬁcult. Clinical observations as well as research results have revealed symptom overlap between ASD and MAD, meaning that symptoms occur in ASD as well as in MAD. Respective symptoms are preoccupations, repetitive behaviors (e.g. obsessions and compulsions), and speech irregularities. Furthermore, individuals with ASD and those with MAD show deficits in emotion recognition (Cath et al., 2008; Collin et al., 2013), avoidance of social situations, withdrawal, impairments in reciprocal social interaction and reduced eye contact (Hartley & Sikora, 2009; Towbin et al., 2005; Tyson & Cruess, 2012; van Steensel et al., 2013), and restricted, repetitive, and stereotyped behaviors (Hartley & Sikora, 2009; Postorino et al., 2017). Emotion regulation, self-regulation, and insistence on sameness are related to anxiety—in individuals with ASD and MAD (Cai et al., 2018; Uljarevic et al., 2017). The level of symptomatology of anxiety and depression in individuals with MAD is positively associated with the presence of autistic traits (Jackson & Dritschel, 2016; Kanne et al., 2009; Liew et al., 2015).

Furthermore, MAD and ASD are difficult to distinguish because of overlapping predictors in early life (Shephard et al., 2019), such as behavioral inhibition representing a strong risk factor for anxiety in later life (Sandstrom et al., 2020), for both internalizing disorders and ASD (Schiltz et al., 2018; Simms, 2017). From this perspective, it is not surprising that children with anxiety disorders (without ASD) score higher for “ASD traits” in early and current development than typically developing children (van Steensel et al., 2013).

In a clinical context, the differentiation between ASD and MAD has particular implications for treatment planning. The most frequently evaluated psychological treatment for anxiety disorders is cognitive behavior therapy (CBT), which involves the application of exposure techniques to enable the individual to endure feared situations accompanied by cognitive restructuring procedures. Misclassification of anxiety disorder as ASD could increase the risk that this individual would not get these effectful interventions on time. In addition, there is evidence that individuals with anxiety disorders with some ASD traits (but not fulfilling a diagnosis of ASD) benefit more from family-oriented CBT compared with individual CBT (Puleo & Kendall, 2011), whereas an adapted CBT is efficacious for individuals with ASD and interfering anxiety (Wood et al., 2020).

In summary, there are a high amount of symptom overlap and high rates of comorbidities between ASD and MAD, leading to a challenging diagnostic process that requires diagnostic instruments with sufficient specificity to plan adequate treatment regimens. To understand the differences between diagnostic categories, disorders are usually compared in individuals without comorbidity to describe single diagnoses among individuals with overlapping symptoms (e.g. Grzadzinski et al., 2011, 2016). With regard to clarification for the diagnostic determination, evidence from previous research has been few and far between on the following questions: “Which behavioral items discriminate best between ASD and MAD?” and “What are the main overlapping symptoms and which behavioral aspects discriminate these disorders?” A better understanding of these questions is most relevant for clinicians, as it might help to guide the diagnostic process and individual treatment planning.

In this study, we thus examined scores of the Autism Diagnostic Observation Schedule (ADOS; Poustka et al., 2015; Rühl et al., 2004) and the Autism Diagnostic Interview–Revised (ADI-R; Bölte et al., 2006). The sample comprised children, adolescents, and adults referred to ASD specialty clinics for a diagnostic evaluation of an ASD suspicion who ultimately received clinical diagnoses of either MAD (ASD was ruled out) or ASD (MAD was ruled out). We aimed to explore (1) the diagnostic accuracy of standard ASD diagnostic instruments, (2) specific profiles of ASD symptoms (on domain and item level) in individuals with MAD compared with individuals with ASD, and (3) the most important items that accurately classify ASD versus MAD cases. To develop an understanding of clearly differentiating features, patients with comorbid ASD and MAD were explicitly ruled out.

## Method

### Participants

Data from the current study were extracted from an established research database of the ASD-Net, a state-funded German research network (Kamp-Becker et al., 2017), which was approved by the ethics committee of the Philipps-University Marburg (AZ: 92/20). The data represent a subsample of patients who have been referred to four specialty outpatient clinics for ASD located in cities and in more rural regions in Germany for diagnostic assessment because of suspicion of ASD. Patients were only eligible for this study if they had received an overall best estimate clinical diagnosis (BEC) of ASD or MAD following a comprehensive diagnostic assessment (see next). Because of a low prevalence rate of MAD in young children in the ASD-Net sample, the analyses were undertaken for patients who were examined with a Module 3 or 4 of the ADOS (see Tables 1 and 2). We aimed to examine a unique clinical sample given that most comparative studies used research rather than clinical samples, mainly including participants with typical development as comparison group. This implies that our study sample, analyses, and results should be particularly relevant for clinicians.

**Table 1.** Frequencies of mood and anxiety disorders included.

Disorder	Module 3 *n* (%)	Module 4 *n* (%)	ADI *n* (%)
Major depressive disorder, single episode (F32)	4 (2.3)	13 (14.6)	3 (4.9)
Major depressive disorder, recurrent (F33)	0 (0.0)	22 (24.7)	5 (8.2)
Persistent mood disorder (F34)	1 (0.6)	5 (5.6)	1 (1.6)
Phobic anxiety disorders (F40)^a^	6 (3.5)	35 (39.3)	4 (6.6)
Other anxiety disorders (F41)	2 (1.2)	1 (1.2)	2 (3.3)
Obsessive-compulsive disorder (F42)	2 (1.2)	5 (5.6)	4 (6.6)
Emotional disorders with onset specific to childhood (F93)	157 (91.3)	8 (9.0)	42 (68.9)

Autism Diagnostic Observation Schedule: *N* = 261 (Module 3: *n* = 172, Module 4: *n* = 89). ADI: *N* = 61. ADI: Autism Diagnostic Interview.

a Predominantly social phobia.

**Table 2.** Mean, standard deviations, and putative between-group differences regarding age, IQ, and IQ level.

Variable	ASD	MAD	Total	*t*-value	*p*
Module 3					
Age	10.3 (2.8)	10.2 (2.5)	10.3 (2.7)	−0.28	0.778
IQ	99.0 (17.8)	99.5 (16.5)	99.2 (17.4)	0.30	0.768
IQ level	3.0 (0.8)	3.0 (0.7)	3.0 (0.8)	−0.20	0.084
Module 4					
Age	24.0 (11.2)	25.1 (10.8)	24.4 (11.1)	0.77	0.444
IQ	105.5 (16.2)	104.7 (14.4)	105.3 (15.7)	−0.34	0.736
IQ level	2.8 (0.8)	2.9 (0.6)	2.8 (0.7)	0.84	0.405
ADI					
Age	13.2 (6.3)	15.0 (10.1)	13.5 (7.0)	1.31	0.193
IQ	99.6 (17.9)	100.0 (22.3)	99.6 (18.6)	0.14	0.381
IQ level	3.0 (0.9)	2.9 (0.8)	3.0 (0.9)	−0.27	0.784

Module 3: *N* = 560. Module 4: *N* = 287. ADI: *N* = 428. IQ: intelligence quotient; ASD: autism spectrum disorder; MAD: mood and anxiety disorder; ADI: Autism Diagnostic Interview.

All patients were diagnosed based on “gold standard” BEC, which rely on the evaluation of two clinicians after extensive examination and review of all information available from the patient’s record (intelligence quotient (IQ), neuropsychological testing, reports from other institutions, school reports, home videos, ADOS, ADI-R, differential diagnostic examination with established structured questionnaires and structural clinical interviews frequently used in German-speaking countries; German Association of the Scientific Medical Societies (AWMF), 2016; National Institute for Health and Clinical Excellence, 2011). Diagnoses were based on the *International Statistical Classification of Diseases and Related Health Problems, 10th Revision* (ICD-10; World Health Organization (WHO), 2004). Within the ASD group, ASD subtypes according to ICD-10 (F84.0, F84.1, and F84.5) were grouped together, whereas the non-ASD group consisted of patients with mood, anxiety, and emotional disorders (MAD, see Table 1). Different MAD diagnoses were grouped together to form a sufficiently large comparison group. Further exploration of different MAD diagnoses was only carried out for specificity analyses.

The study included data of 847 participants (*n* = 586 with ASD, *n* = 261 with mood, anxiety, or emotional disorders), all evaluated with the ADOS in the context of the diagnostic process. The sample was separated for the identification of the most discriminative behavioral items insofar as individuals were administered different measures (i.e. ADOS modules) depending on age and language ability. The subgroups are henceforth labeled Module 3 (children and younger adolescents, fluent language) and Module 4 (adolescents and adults, fluent language). A total of 560 participants were tested with Module 3 (*n* = 388 with ASD, 8.8% female, 5–22 years of age, *median age* = 10.0, *interquartile range* (*IQR*) = 4; *n* = 172 with MAD, 18.6% female, 5–17 years of age, *median age* = 10.0, *IQR* = 4), whereas 287 individuals were tested with Module 4 (*n* = 198 with ASD, 26.3% female, 13–65 years of age, *median age* = 19.5, *IQR* = 14; *n* = 89 with MAD, 36.0% female, 13–53 years of age, *median age* = 20.0, *IQR* = 17). Complete ADI-R data were available for 428 participants (5–51 years of age, *n* = 367 with ASD, 12.0% female; *n* = 61 with MAD, 16.4% female).

#### Community involvement

There was no community involved.

### Measures

The German Versions of the ADOS (ADOS-G and ADOS-2; Poustka et al., 2015; Rühl et al., 2004) and the ADI-R (Bölte et al., 2006) were administered to obtain information about ASD symptoms. The ADOS is a semi-structured and standardized observation tool which is part of the established gold standard to diagnose ASD (German Association of the Scientific Medical Societies (AWMF), 2016; National Institute for Health and Clinical Excellence, 2011). The ADOS consists of four modules (plus a toddler module in ADOS-2), one of which is selected depending on the age and verbal skills of a participant. It comprises a semi-structured interaction of the participant with a clinically trained administrator to capture important social communicative behaviors as well as stereotypic and repetitive behavioral features. Module 3 is intended for verbally fluent children and younger adolescents and Module 4 is intended for verbally fluent adolescents and adults. In Module 3, relevant aspects are coded with the help of 29 items. In Module 4, 31 (ADOS-G), or 32 (ADOS-2), items have to be coded. The additional item of ADOS-2 (“Amount of Social Overtures/Maintenance of Attention”) was not included into the analyses because the data set was based on ADOS-G and ADOS-2. Codes fall on an ordinal scale from 0 (*no abnormality related to autism*), 2 (*definite evidence of abnormality*) to 3 (*profound severity*), with additional codes of 7 and 8 for abnormal behavior or behavior not exhibited during the observation, and a code of 9 for missing values (i.e. answers omitted or left blank). The behavioral items were grouped into two domains: social affect (SA) and restricted and repetitive behavior (RRB). The ADOS diagnostic algorithms yield classifications of autism and autism spectrum versus non-ASD.

The ADOS is usually combined with the Autism Diagnostic Interview–Revised (ADI-R), a standardized, semi-structured clinical interview for caregivers of individuals suspected of having ASD. Responses are scored by trained administrators based on the caregiver’s description of the child’s behavior. The ADI-R yields three domain scores based on behavior (between the ages of 4 and 5, or ever), which are calculated by summing items within the areas of qualitative abnormalities in reciprocal social interaction (social), qualitative abnormalities in communication (communication), and restricted, repetitive, and stereotyped patterns of behavior (RRB). Totals from domains can be compared with algorithm cutoffs that yield classifications of autism or nonautism.

### Data preparation

For data preparation, ADOS and ADI-R codes of 7 and 8 were recoded to 0, and domain scores were calculated as suggested in the ADOS and ADI-R manuals after codes of 3 were recoded to 2. For Module 3, the ADOS-2 algorithm was used to calculate scores. For Module 4, the revised algorithm of ADOS-2 (Hus & Lord, 2014) and the algorithm of ADOS-G were used, as the ADOS-G algorithm is included in the ADOS-2 manual. To be able to compare values of Module 3 and Module 4, it is possible to compute Calibrated Symptom Severity Scores (CSS), which are comparable through all modules (Hus et al., 2014; Hus & Lord, 2014). Thus, domain comparisons were calculated with codes of 0 to 2 and recoded into CSS (for ADOS), whereas analyses on item level and regression analyses were computed with codes from 0 to 3. ADOS and ADI-R data only included participant data without missing with regard to items.

### Statistical analyses

To explore the diagnostic accuracy of standard ASD diagnostic instruments (Aim 1), ADOS algorithm cutoff scores, domain scores for SA, RRB, and the total calibrated severity scores (CSS) were calculated for the full sample. Based on the algorithm cutoffs, percentages of MAD and ASD cases meeting diagnostic thresholds of the ADOS and ADI-R were computed. Group differences on domain levels of ADOS (CSS Total, CSS SA, CSS RRB) and ADI-R (social, communication, and RRB) were tested by analyses of variance.

To identify specific profiles of ASD symptoms on domain and item level in individuals with MAD compared with individuals with ASD (Aim 2), we first identified items that discriminated best between ASD and MAD by a methodology for discrimination purposes (along the lines of Gotham et al., 2007; Grzadzinski et al., 2013). Following Grzadzinski and colleagues (2013), an item was determined to be “adequately” discriminative if it was endorsed (Codes 1–3) in more than 66% of the ASD group and less than 33% in the MAD group.

To determine the most important items suitable for accurate classification of ASD versus MAD cases (Aim 3) in the full sample, we performed an ensemble feature selection (EFS; for detailed description, see Neumann et al., 2017). This method combines different feature selection methods to compensate for biases of single methods. Feature selection consists of selecting the relevant features for a problem and discard those irrelevant or redundant, with the main goal of improving classification accuracy (Neumann et al., 2017; Remeseiro & Bolon-Canedo, 2019). Thus, results of EFS will give an overview of the relevance of every item. We performed this analysis with the data set including complete ADOS and ADI-R data (*N* = 334; 281 ASD cases and 53 MAD cases).

**Figure 1.**
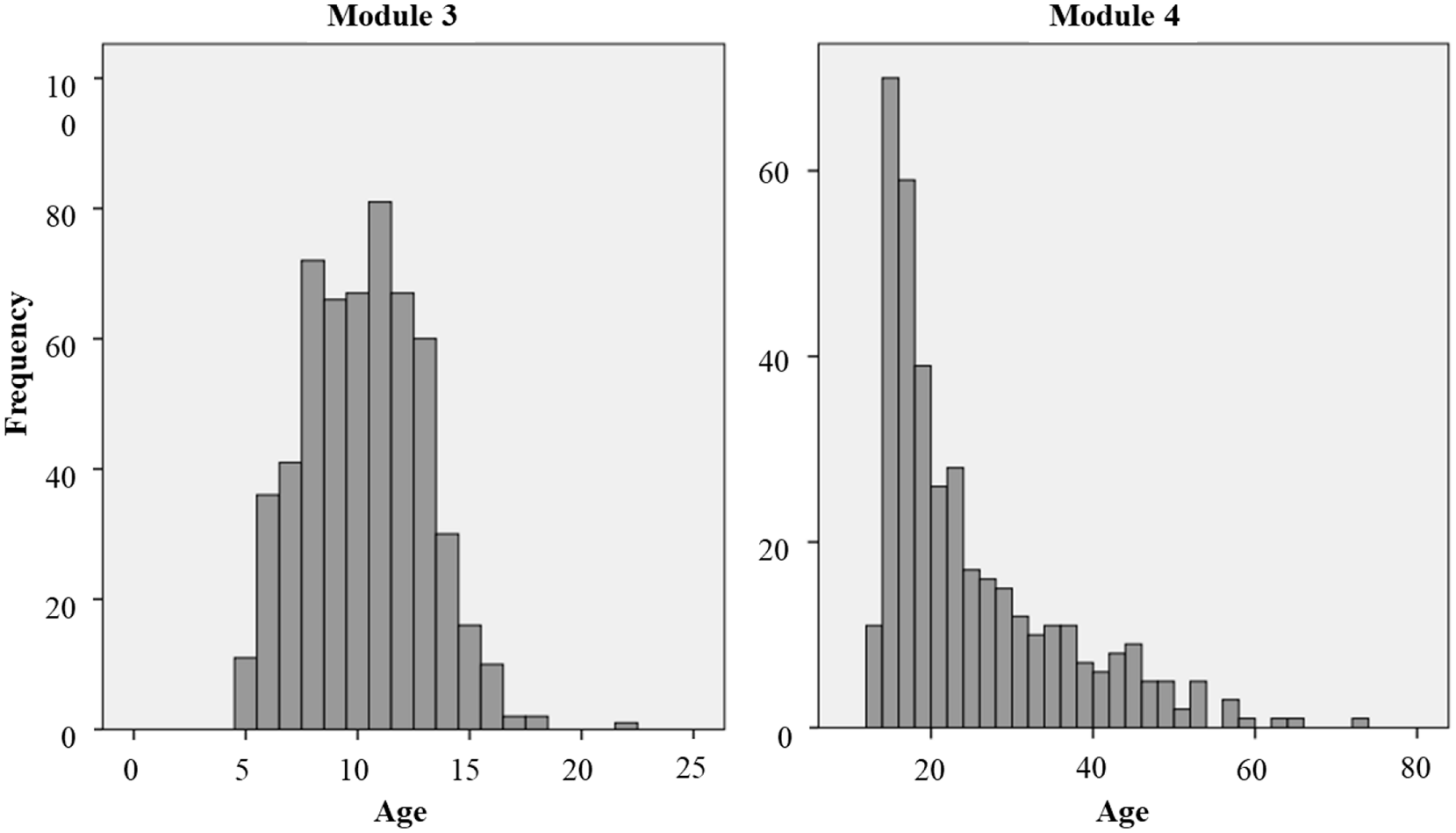
Distribution of age. The histogram shows the frequencies of ages included in the samples of Modules 3 and 4.

Subsequently, in a separate analysis, binominal logistic regressions were computed on two subsamples as the samples of ADOS Modules 3 and 4 differ widely concerning age (see Figure 1). Regression analyses were computed to identify those ADOS and ADI-R items which were able to discriminate between ASD and MAD in our two subsamples. For both ADOS and ADI-R, logistic regressions were computed with a maximum likelihood estimator. The selection method was forward selection with Wald statistics. This selection method combines a test of inclusion with significance testing of the item score (*p*-value for inclusion < 0.01) and a test of exclusion based on Wald statistics, following a chi-square distribution (*p*-value for exclusion > 0.05 for the entire model fit). Thus, items were added to the regression model, if they lay under the cutoff for exclusion and if a significant improvement in the model is achieved.

## Results

Table 1 shows the frequencies of specific MAD disorders included into our MAD sample. In the ASD group, comorbid MAD disorders were an exclusion criterion, whereas other comorbid disorders such as ADHD were not.

Complete IQ data were available for 690 participants (81.5% of the entire sample), whereas IQ-level data (following ICD-10; taken from previous investigations, documented in medical letter) were available for 753 participants (88.9% of the entire sample). Preliminary analyses on significant group differences on age, IQ, and IQ level are reported in Table 2. There were no group differences regarding age or IQ in Module 3, Module 4, or ADI-R. In addition, median age and IQR did not differ within the modules, so these variables (age and IQ) were not considered as confounding variables in the subsequent analyses.

### Diagnostic accuracy of ADOS and ADI-R

In Module 3, 16.9% of the MAD sample (*n* = 29) met the autism spectrum cutoff, whereas 7.0% (*n* = 12) met the autism cutoff. In Module 4, 16.9% (*n* = 15) met the autism spectrum cutoff and 9.0% (*n* = 8) met the autism cutoff of the revised algorithm. For the ADOS-G algorithm of Module 4, 19.1% (*n* = 17) met the autism spectrum cutoff, whereas 7.9% (*n* = 7) met the autism cutoff.

To explore whether there was a specific subgroup of the MAD sample that exceeded the thresholds, we divided the MAD sample into a mood disorder subgroup (F32–F34 according ICD-10, *n* = 45), an anxiety disorder subgroup (F40–F41, *n* = 44), and an emotional disorder with onset specific to childhood group (F93, *n* = 165). About 25.0% of the anxiety disorder group, 17.8% of the mood disorder group, and 15.2% of the emotional disorder group met ADOS spectrum cutoffs. In contrast, 88.9% of the ASD sample in Module 3 (ADOS-2) and 80.3% of Module 4 (revised algorithm) met autism spectrum cutoffs. Investigations of the ADI-R showed that 34.4% of the MAD sample met algorithm criteria for the reciprocal social interaction domain, 18% for the communication, and 26.2% for the RRB domain. In total, 49.2% of MAD cases met at least one out of three ADI-R thresholds, 19.6% fulfilled two, and 9.8% all three thresholds. However, only one of the six participants who fulfilled all three thresholds of the ADI-R also met the ADOS autism spectrum algorithm cutoff.

### Domain comparison

**Figure 2.**
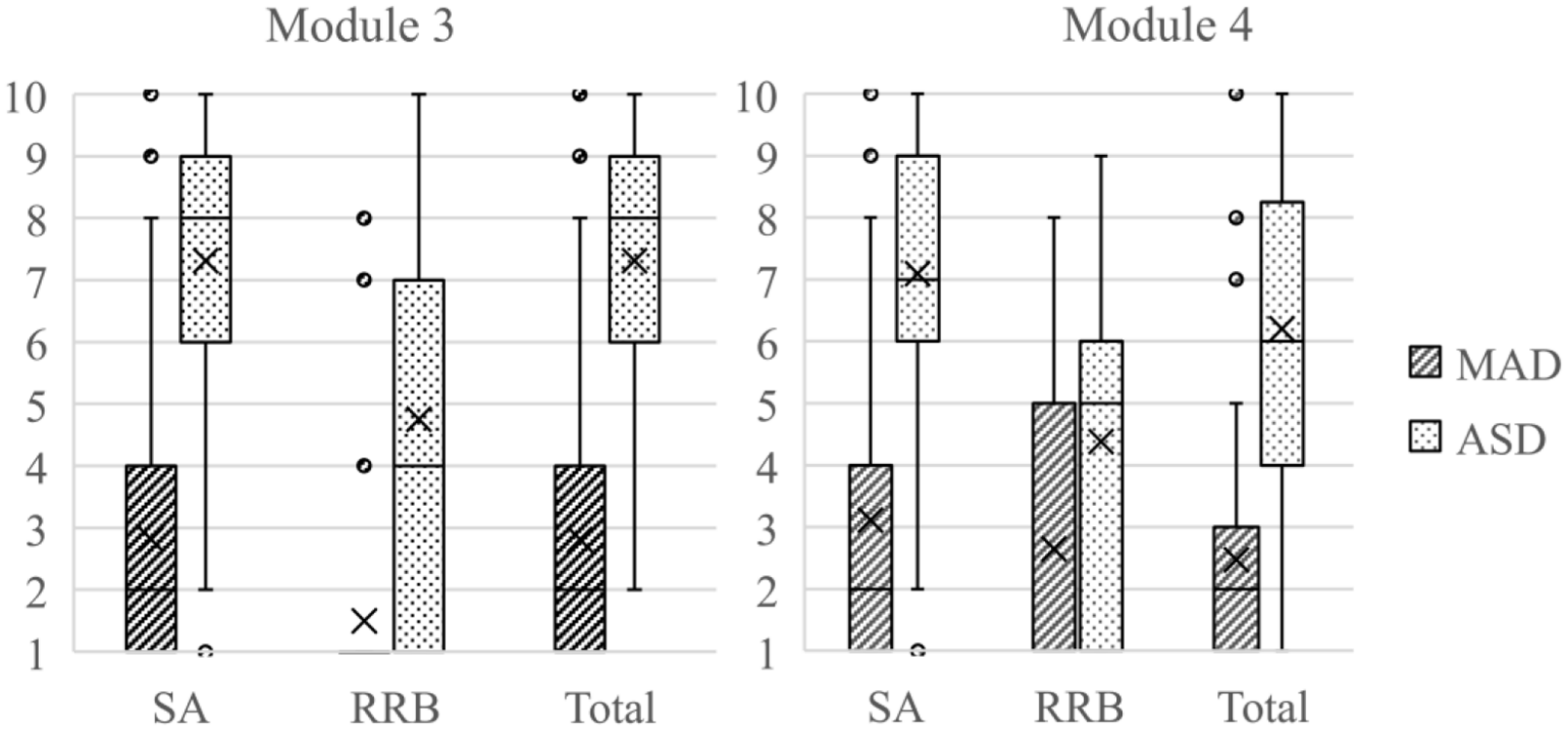
Boxplot for CSS scores of the ADOS. The boxplot shows quartile range. Middle lines indicate medians. An X indicates the group’s mean. Dots indicate outliers. SA: social affect; RRB: restricted, repetitive behavior; total: total score (sum of SA + RRB); CSS: calibrated severity score; ADOS: Autism Diagnostic Observation Schedule.

Figure 2 shows distributions of algorithm scores of the ADOS in the form of CSS for ASD and MAD. Not surprisingly, the ASD group scored significantly higher on all CSS domains. For Module 3, CSS Overall: *F*(1, 558) = 503.82, *p* < 0.001, η^2^ = 0.47; CSS SA: *F*(1, 558) = 491.5, *p* < 0.001, η^2^ = 0.47; and CSS RRB: *F*(1, 558) = 202.7, *p* < 0.001, η^2^ = 0.27. For Module 4, CSS Overall: *F*(1, 285) = 157.3, *p* < 0.001, η^2^ = 0.36; CSS SA: *F*(1, 258) = 206.5, *p* < 0.001, η^2^ = 0.42; and CSS RRB: *F*(1, 285) = 37.8, *p* < 0.001, η^2^ = 0.12. As illustrated in Figure 2, there are some outliers and an overall high variance in the symptom severity.

**Figure 3.**
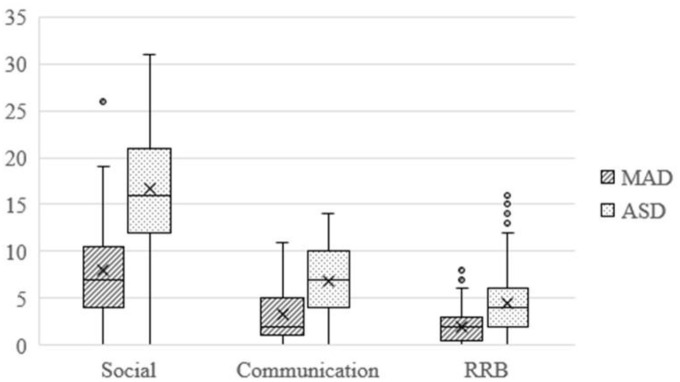
Boxplot for algorithm scores of ADI-R. It shows quartile range. Middle lines indicate medians. An X indicates the group’s mean. Dots indicate outliers. Social: social domain score; communication: communication domain score; RRB: restricted, repetitive behavior domain score; ADI-R: Autism Diagnostic Interview–Revised.

Algorithm scores for ADI domains were presented in Figure 3. Again, the ASD group scored higher on all ADI domains: social: *F*(1, 426) = 103.2, *p* < 0.001, η^2^ = 0.20; communication: *F*(1, 426) = 57.81, *p* < 0.001, η^2^ = 0.12; and RRB: *F*(1, 426) = 39.28, *p* < 0.001, η^2^ = 0.08.

### Comparison on item level

Six items of Module 3 (see Table 3) met criteria of adequate discrimination between ASD and MAD. In addition, six items of Module 4 (see Table 3) met criteria to be adequately discriminative between ASD and MAD. For ADI-R algorithm, only one item, Imitative Social Play, met criteria to discriminate adequately between the ASD and MAD groups (endorsed in 76.8% of ASD and 29.5% of MAD).

**Table 3.** Items that met criteria for adequate distinction between MAD and ASD.

ADOS Items Module 3	MAD *n* (%)	ASD *n* (%)
Communication domain		
Speech abnormalities associated with autism	44 (25.6)	298 (76.7)
Reporting of events^a^	53 (30.8)	295 (77.2)
Conversation^a^	51 (29.7)	323 (83.3)
Reciprocal social interaction domain		
Facial expressions directed to examiner^a^	50 (29.1)	330 (85.1)
Quality of social overtures^a^	41 (23.8)	341 (87.9)
Quality of social response^a^	56 (32.6)	301 (77.6)
ADOS Items Module 4	MAD *n* (%)	ASD *n* (%)
Communication domain		
Conversation^a^	25 (28.1)	143 (72.2)
Descriptive, conventional, instrumental, or informational gestures^a^	25 (28.1)	145 (73.2)
Reciprocal social interaction domain		
Insight^b^	29 (32.6)	160 (80.8)
Quality of social overtures^a^	19 (21.4)	146 (73.7)
Quality of social response^a^	23 (25.8)	144 (72.7)
Restricted repetitive behaviors		
Speech abnormalities associated with autism^b^	28 (31.5)	136 (68.7)

*N* = 847. Module 3: *n* = 560. Module 4: *n* = 287. Items met criteria for adequate distinction between groups if they were endorsed in >66% of ASD and <33% of MAD. MAD: mood and anxiety disorder; ASD: autism spectrum disorder; ADOS: Autism Diagnostic Observation Schedule.

a Algorithm items of ADOS-2. ^b^Not included in ADOS but in ADOS-2 in Module 4.

### Logistic regression

The model based on ADOS items of Module 3, identified by logistic regression, explained 76.8% of the variance. In addition, the percentage of accuracy in classification (PAC) was high with PAC = 91.3% (MAD = 85.5%, ASD = 93.8%). Six items were included into the model indicating to be discriminative (see Table 5), of which two had been identified to be discriminative based on the proportion of endorsed item values.

For Module 4, the logistic regression model explained 50.6% of the variance. The PAC was lower than for Module 3 with 83.6% (MAD = 75.3%, ASD = 87.4%). Five items were included into the model (see Table 4). One item, which was identified to be discriminative based on the proportion of endorsed item values, was also included into the regression model.^1^

**Table 4.** Results of logistic regression on item level of ADOS.

ADOS Module 3 (*N = 560, df = 1*)				
Variable (domain)	*B*	Wald	*p*	Odds ratio
Stereotyped/idiosyncratic use of words or phrases^a^ (RRB)	2.81	29.80	0.000	16.56
*Quality of social overtures*^a^ (Social)	1.48	27.24	0.000	4.41
*Conversation*^a^ (Com)	1.23	20.48	0.000	3.43
Insight (Social)	1.04	22.15	0.000	2.83
Descriptive, conventional, instrumental, or informational gestures^a^ (Com)	0.92	14.41	0.000	2.52
Anxiety (Other abnormal behavior)	−2.06	32.35	0.000	0.13
Constant	−2.82	59.40	0.000	0.06
ADOS Module 4 (*N* *=* *287, df* *=* *1*)				
Amount of reciprocal social communication^a^ (Social)	1.66	22.97	0.000	5.26
*Insight*^a^ (Social)	0.86	11.54	0.001	2.35
Empathy/comments on other’s emotions (Social)	0.84	9.94	0.002	2.32
Unusual eye contact^a^ (Social)	0.51	7.62	0.006	1.67
Offers information (Com)	−1.12	9.16	0.002	0.33
Constant	−1.76	31.58	0.000	0.17

In Italics: *>*66% ASD and *<*33% MAD. Odds ratios >1.00 indicate increasing likelihood of ASD, whereas values <1.00 indicate increasing likelihood of being assigned to MAD. ADOS: Autism Diagnostic Observation Schedule; Com: communication domain; Social: social interaction domain; RRB: restricted repetitive behaviors; ASD: autism spectrum disorder; MAD: mood and anxiety disorder.

a Algorithm items.

Logistic regression was also calculated for ADI-R algorithm items, resulting in a model explaining 35.5% of the variance. Total PAC was 87.8% by better PAC for ASD (97.5%) than for MAD (29.5%). Five ADI-R items were identified to discriminate between ASD and MAD (see Table 5).

**Table 5.** Results of logistic regression on item level of ADI-R.

Variable (subdomain)	*B*	Wald	*p*	Odds ratio
Hand and finger mannerisms (C3)	1.04	8.49	0.004	2.83
Unusual preoccupations (C1)	0.78	7.92	0.005	2.18
Reciprocal conversation (B2)	0.65	7.60	0.006	1.91
Interest in children (A2)	0.63	8.89	0.003	1.88
Group play with peers (A2)	0.60	7.65	0.006	1.83
Constant	−0.88	7.35	0.007	0.42

*N* = 426 (ASD = 365, MAD = 61). *df* = 1. Odds ratios *>*1.00 indicate increasing likelihood of ASD, whereas values *<*1.00 indicate increasing likelihood of being assigned to MAD. Social Interaction domain, subdomain A2: failure to develop peer relations; communication domain, subdomain B2: relative failure to initiate or sustain conversational interchange; restricted, repetitive, and stereotyped pattern of behavior domain, subdomain C1: encompassing preoccupation or circumscribed pattern of interest; C3: stereotyped and repetitive motor mannerisms; ADI-R: Autism Diagnostic Interview–Revised; ASD: autism spectrum disorder; MAD: mood and anxiety disorder.

### EFS

**Figure 4.**
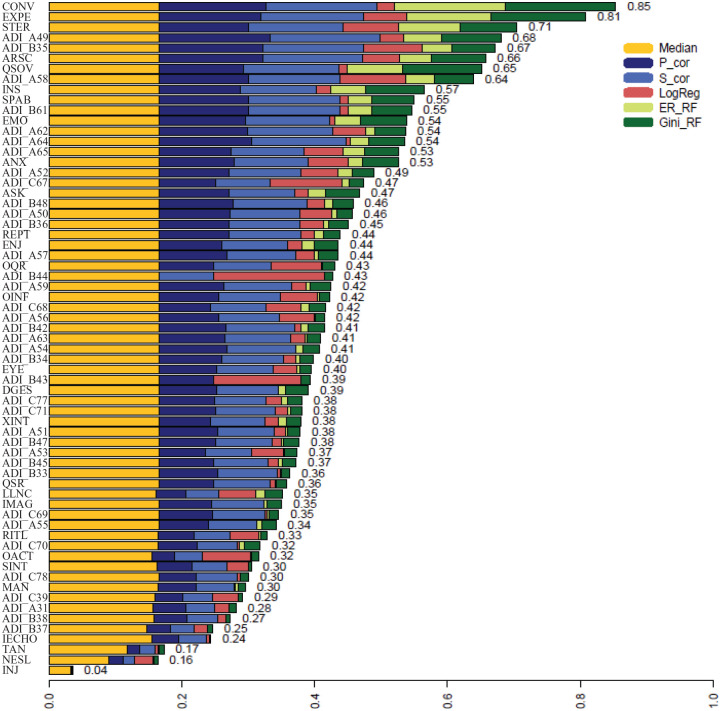
Results of ensemble feature selection. Values report item importance with higher values indicating higher importance for classification. Median: *p*-values from Wilcoxon signed-rank test; P_cor: Pearson product-moment correlation; S_cor: Spearman rank correlation; LogReg: β-values of logistic regression; ER_RF: error-rate based on random Forest analysis; Gini_RF: Gini-index based on random Forest analysis; ANX: anxiety; ARSC: amount of reciprocal social communication; ASK: asks for information; CONV: conversation; DGES: descriptive, conventional, instrumental, or informational gestures; EMO: empathy/comments on other’s emotions; ENJ: shared enjoyment in interaction; EXPE: facial expressions directed to examiner; EYE: unusual eye contact; IECHO: immediate echolalia; IMAG: imagination/creativity; INJ: self-injurious behavior; INS: insight; LLNC: language production and linked nonverbal communication; MAN: hand and finger and other complex mannerisms; NESL: overall level of nonechoed language; OACT: overactivity; OINF: offers information; OQR: overall quality of rapport; QSOV: quality of social overtures; QSR: quality of social response; REPT: reporting of events; RITL: compulsions or rituals; SINT: unusual sensory interest in play material/person; SPAB: speech abnormalities associated with autism; STER: stereotyped/idiosyncratic use of words or phrases; TAN: tantrums, aggression, negative or disruptive behavior; XINT: excessive interest in or references to unusual or highly specific topics or objects or repetitive behaviors; ADI_A31: use of other’s body to communicate; ADI_A49: imaginative play with peers; ADI_A50: direct gaze; ADI_A51: social smiling; ADI_A52: showing and directing attention; ADI_A53: offering to share; ADI_A54: seeking to share enjoyment with others; ADI_A55: offering comfort; ADI_A56: quality of social overtures; ADI_A57: range of facial expressions used to communicate; ADI_A58: inappropriate facial expressions; ADI_A59: appropriateness of social response; ADI_A62: interest in children; ADI_A63: response to approaches of other children; ADI_A64: group play with peers; ADI_A65: friendships; ADI_B33: stereotyped utterances and delayed echolalia; ADI_B34: social verbalization/chat; ADI_B35: reciprocal conversation; ADI_B36: inappropriate questions of statements; ADI_B37: pronominal reversal; ADI_B38: neologism/idiosyncratic language; ADI_B42: pointing to express interest; ADI_B43: nodding; ADI_B44: head shaking; ADI_B45: conventional/instrumental gestures; ADI_B47: spontaneous imitation of actions; ADI_B48: imaginative play; ADI_B61: imitative social play; ADI_C39: verbal rituals; ADI_C67: unusual preoccupations; ADI_C68: circumscribed interests; ADI_C69: repetitive use of objects or interest in parts of objects; ADI_C70: compulsions/rituals; ADI_C71: unusual sensory interests; ADI_C77: hand and finger mannerisms; ADI_C78: other complex mannerisms or stereotyped body movements.

The results of the EFS (Neumann et al., 2017) are presented in Figure 4. In addition, the features with the highest importance scores using the EFS to predict ASD diagnoses derived from ADOS and ADI are listed in Table 6. There are two items with exceedingly high feature importance for the discrimination between ASD and MAD. Another six items formed a second block in the EFS results. Using only the first two top-rated items of the EFS in the prediction of ASD or MAD resulted in an area under the curve (AUC) of over 83%. Using the first eight items resulted in an AUC of 94%. With a cutoff of 5, the sensitivity of the eight items was .91, while specificity was .89. The items Insight and Empathy of the ADOS, which we identified by regression analysis, were in the third block of important features, with slightly reduced importance compared with the first eight items.

**Table 6.** Results of EFS: The first eight ADOS and ADI-R items with highest importance value.

Item	Diagnostic instrument	Importance value
Conversation	ADOS (Com)	0.85
Facial expressions directed to examiner	ADOS (Social)	0.81
Stereotyped/idiosyncratic use of words or phrases	ADOS (RRB)	0.71
Imaginative play with peers	ADI-R (Social)	0.68
Reciprocal conversation	ADI-R (Com)	0.67
Amount of reciprocal social communication	ADOS (Social)	0.66
Quality of social overtures	ADOS (Social)	0.65
Inappropriate facial expressions	ADI-R (Social)	0.64

Importance value indicates importance of item in terms of classification of ASD and MAD. Com: communication domain; Social: social Interaction domain; RRB: restricted repetitive behaviors; EFS: ensemble feature selection; ADOS: Autism Diagnostic Observation Schedule; ADI-R: Autism Diagnostic Interview–Revised; ASD: autism spectrum disorder; MAD: mood and anxiety disorder.

## Discussion

Due to high rates of comorbidities and a wide symptom overlap with other psychiatric conditions, the (differential-) diagnostic process of ASD in clinical settings can be challenging. This is particularly true for the assessment of verbally fluent children, adolescents, and adults with multiple social and behavioral problems. Thus, more finely tuned and precise information about the symptom overlap and differences between ASD and other mental disorders such as MAD is needed to create a better understanding for diagnostic purposes as well as tailoring individualized treatment strategies. We explored ASD symptoms in patients with MAD who were initially referred to ASD specialized clinics with suspicion of ASD and thus represent a well-balanced and most relevant clinical sample. By examining those behavioral aspects that best discriminate between ASD and MAD, this study contributes substantially to the understanding of differential diagnoses in ASD and highlights the need to be cautious in overestimating the diagnostic utility of the established instruments to support diagnostic decision-making in clinical samples with symptoms of mood, anxiety, and emotional disorders.

### Diagnostic accuracy

Several patients of the MAD group showed ASD symptoms to an extent exceeding the diagnostic thresholds of the instruments. This indicates a high amount of symptom overlap between these two disorders. Given the fact that the examined participants were referred to specialized ASD clinics due to ASD concerns, this result is not surprising and consistent with previous studies showing that the disorders share a substantial number of symptoms (Collin et al., 2013; Towbin et al., 2005; van Steensel et al., 2013). In our sample, this was especially the case for social anxiety disorders.

In our study, percentages exceeding ADI-R threshold were high for MAD participants, compared with those reported in a study by van Steensel and colleagues (2013). Furthermore, we found an extensive overlap in the domain of social interaction, whereas van Steensel and colleagues (2013) found the greatest symptom overlap in the domain of repetitive behavior. These differences might be due to differences in the sample characteristics, as the sample of van Steensel and colleagues (2013) was much smaller, but also significantly younger. Differences in symptom overlap could indicate the presence of common developmental pathways or convergence in early behavioral manifestations of these disorders (Shephard et al., 2019). Another fundamental difference is that participants of our MAD sample had a suspicion of ASD, whereas none of the children in the sample of van Steensel et al. (2013) was suspected of having ASD. This might indicate that especially deficits in the domain of social interaction may lead to the suspicion of ASD.

In line with Sikora and colleagues (2008), nearly 17% of our MAD sample exceeded the thresholds of the ADOS. Participants with anxiety disorders showed the highest percentage above ADOS cutoff, whereas Sikora and colleagues (2008) found the highest percentage (29.4%) in those with mood disorder (13.6% for anxiety disorders). Again, the sample of Sikora and colleagues (2008) was much smaller and significantly younger than our sample.

### Specific profiles of ASD symptoms

#### Domain level

The ADOS performed slightly better than the ADI-R, resulting in more items identified to adequately discriminate between the MAD and ASD groups. On the basis of a more clinical-methodological approach (differences in domain scores, criteria for adequate distinction), our results indicate that the ASD group scored significantly higher on all domain scores of the ADOS and the ADI-R, despite high variance, outliers and low to medium effect sizes.

The number of ADOS (algorithm) items that met criteria for adequate distinction between groups was small. Furthermore, there is an outstanding qualitative difference in symptom overlap between ASD and MAD, as examined in our study, compared with results on ASD versus ADHD by Grzadzinski and colleagues (2016). The observation that most items of a diagnostic tool developed to identify autism are also endorsed for other disorders requires careful consideration. Our results reveal the great amount of symptom overlap between ASD and MAD, which requires a better understanding to reliably distinguish both disorders.

#### Item level

Results of regression analyses indicate that, especially for Module 3, a satisfactory amount of variance could be explained by the identified model with high percentages of accuracy in classification. Besides already mentioned discriminating items (quality of social overtures, conversation), we found that the use of stereotyped words or phrases is a strong indicator for ASD. Abnormalities in insight and gestures also increase probability of ASD. Reduced reciprocal social communication and abnormalities in insight and empathy are indicators for ASD, whereas abnormalities in offering information increase the probability of MAD in adolescents and adults. These results indicate that a combination of communicational and social cognition aspects is relevant for the discrimination between ASD and MAD, while many other items overlap in ASD and MAD. Of course, the observation of anxiety-related symptoms—including initial wariness or self-consciousness as well as more obvious signs of worry, upset or concern—is a significant indicator for MAD and should thus receive close attention during administration of the ADOS. Our results indicate that retrospective information from caregivers alone, as collected by the ADI-R, is not appropriate for the discrimination of ASD and MAD.

#### EFS

By using an EFS approach, a quantification of the importance of all single features of the ADOS in combination with the ADI-R could be obtained. A combination of eight items (five from the ADOS and three from the ADI-R) could be identified to increase both sensitivity and specificity. It seems that the combination of these instruments is essential to accurately classify ASD versus MAD.

The gold standard in ASD diagnostic processes is the combination of a behavioral observation through the ADOS, which is conducted via one-on-one interaction and observation for a limited time period and, at a present time point, with an extensive determination of anamnestic data (German Association of the Scientific Medical Societies (AWMF), 2016; National Institute for Health and Clinical Excellence, 2011). Anamnestic data are very important, as one critical criterion for ASD is the presence of symptoms in the early developmental period. However, information derived from reports on behavior dating back years is vulnerable to several biases that may reduce validity of retrospective statements of caregivers over a long time period (Hus et al., 2014; Jones et al., 2015; Ozonoff et al., 2011).

In accordance with other studies (Guttmann-Steinmetz et al., 2010; Hartley & Sikora, 2009; Pine et al., 2008; Postorino et al., 2017; van Hulle et al., 2012; van Steensel et al., 2013), we found that the presence of repetitive, stereotyped behavior at present (ADOS) and during development (ADI-R)—with the exception of stereotyped or idiosyncratic use of phrases—are not specific for ASD and do not discriminate ASD from MAD.

This selected number of items that show significant discrimination between ASD and MAD could be—in addition to be considered within an ASD diagnostic evaluation—the foundation for the development of a specific screening instrument with training tools for clinicians to evaluate indications of ASD (Stroth et al., 2021).

### Limitations

One limitation of this work is that the substantial sample size still did not allow for differentiation between different mood and anxiety subcategories. Thus, more finely tuned information on even more subtle differences between the different symptomatology could not be derived and should be subject to future investigations. Our sample included individuals with MAD with suspicion of ASD, which is not representative for all individuals with MAD. In addition, as individuals with comorbid ASD and MAD were explicitly excluded from the analyses, results cannot be transferred to this subgroup. Therefore, future studies should investigate clear features that characterize this patient group of comorbid ASD and MAD. Furthermore, although the sample comprises an expectable number of female cases, separate analyses for female individuals, which would be desirable from both a clinical and a theoretical perspective, did not seem appropriate. In addition, there may be confounders that were not considered in our analyses like the child’s intellectual as well as adaptive functioning, comorbidities, and changes in anxiety across development. There is evidence that these factors affect the development and intensity of MAD symptomatology also in individuals with ASD (Ben-Itzchak et al., 2020; Schiltz & Magnus, 2021). Therefore, future investigations should include samples comprising a broader range of thoroughly surveyed data on symptomatology and should also consider confounders like age and IQ, allow for gender differentiations as well as for other potential confounders such as comorbidity or other medical conditions. Furthermore, the eight items found via EFS will be worth a review in an independent sample.

## Conclusion

Our results suggest that—similar to Grzadzinski and colleagues (2016) for ADHD—communicative and social problems are not specific to ASD. Therefore, interventions to address these difficulties should not only depend on an ASD diagnosis. Care must be taken with regard to over-hasty diagnostic decisions, as the degree of overlap between MAD and ASD may result in misinterpretation of symptoms and in high scores in the ADOS and the ADI-R, respectively. In clinical practice, the ADOS and ADI-R gather much more information than is depicted in the scores and cutoffs. In some cases, it can be appropriate to put less emphasis on the scores and cutoffs, and instead use the clinical information obtained to “map onto” the *Diagnostic and Statistical Manual of Mental Disorders* (5th ed.; *DSM*-5) or ICD-10 diagnostic criteria. Caution is required if the clinician observes symptoms of anxiety (including wariness, as well as more obvious signs of worry or concern, as indicated in Item E3 of the ADOS). A differential diagnostic examination with respect to any anxiety or mood disorder has to be undertaken to decide whether observed social impairment or repetitive, stereotyped behavior is explainable through the diagnosis of MAD or clearly meets diagnostic criteria for an additional ASD diagnosis.

There are several further clinical conclusions that result from our study. First, the differentiation of ASD and MAD is difﬁcult, and ADOS and ADI-R items are important to master the challenge. Second, when symptoms of MAD are evident in patients with marked deficits in the domain of social interaction, timely interventions have to address both symptoms. In some cases, the symptoms of ASD and MAD are overlapping to such an extent that it is necessary to start interventions targeting symptoms of MAD. CBT is an effective treatment not only for patients with MAD (James et al., 2015) but also—with minimal adaptations—for those with a diagnosis of ASD (Kreslins et al., 2015; Sukhodolsky et al., 2013; Warwick et al., 2017; Wood et al., 2020). As we know from comprehensive research, comorbid MAD in individuals with ASD can increase symptom levels and reduce the outcome expectancies of ASD (Avni et al., 2018; Hallett et al., 2012; Kamp-Becker et al., 2009; McVey et al., 2018; Spiker et al., 2012; Sukhodolsky et al., 2008). Thus, it is of primary importance to reduce burden and harm of the patients and their families via effective intervention before a diagnostic classification can be made as to whether the MAD symptoms are comorbid to an ASD or the social deficits are comorbid to MAD.

## Notes

1 Regression analyses were not adjusted for potential confounders.
